# An *in vitro *study investigating the survival and phenotype of mesenchymal stem cells following injection into nucleus pulposus tissue

**DOI:** 10.1186/ar2611

**Published:** 2009-02-11

**Authors:** Christine L Le Maitre, Pauline Baird, Anthony J Freemont, Judith A Hoyland

**Affiliations:** 1 Biomedical Research Centre, Biosciences, Faculty of Health and Wellbeing, Sheffield Hallam University, City Campus, Owen Building, Howard Street, Sheffield S1 1WB, UK; 2 Tissue Injury and Repair Group, School of Clinical and Laboratory Sciences, Faculty of Medical and Human Sciences, Stopford Building, The University of Manchester, Oxford Road, Manchester M13 9PT, UK

## Abstract

**Introduction:**

The decreased disc height characteristic of intervertebral disc (IVD) degeneration has often been linked to low back pain, and thus regeneration strategies aimed at restoring the disc extracellular matrix and ultimately disc height have been proposed as potential treatments for IVD degeneration. One such therapy under investigation by a number of groups worldwide is the use of autologous mesenchymal stem cells (MSCs) to aid in the regeneration of the IVD extracellular matrix. To date, however, the optimum method of application of these cells for regeneration strategies for the IVD is unclear, and few studies have investigated the direct injection of MSCs alone into IVD tissues. In the present article, we investigated the survival and phenotype of human MSCs, sourced from aged individuals, following injection into nucleus pulposus (NP) tissue explant cultures.

**Methods:**

Human MSCs extracted from bone marrow were expanded in monolayer culture and, after labelling with adenoviral vectors carrying the green fluorescent protein transcript, were injected into NP tissue explants (sourced from bovine caudal discs) and maintained in culture for 2, 7, 14 and 28 days post injection. Following fixation and paraffin embedding, cell viability was assessed using *in situ *hybridisation for polyA-mRNA and using immunohistochemistry for caspase 3. Immunohistochemistry/fluorescence for aggrecan, Sox-9 and types I, II and X collagen together with Alizarin red staining was employed to investigate the MSC phenotype and matrix formation.

**Results:**

MSCs were identified in all injected tissue samples and cell viability was maintained for the 4 weeks investigated. MSCs displayed cellular staining for Sox-9, and displayed cellular and matrix staining for aggrecan and type II collagen that increased during culture. No type I collagen, type X collagen or Alizarin red staining was observed at any time point.

**Conclusions:**

MSCs from older individuals differentiate spontaneously into chondrocyte-like NP cells upon insertion into NP tissue *in vitro*, and thus may not require additional stimulation or carrier to induce differentiation. This is a key finding, as such a strategy would minimise the level of external manipulation required prior to insertion into the patient, thus simplifying the treatment strategy and reducing costs.

## Introduction

Approximately 11 million people in the UK experience low back pain (LBP) for at least 1 week each month, leading to a considerable loss of working days and significantly impacting on the National Health Service [1,2]. The causes of LBP are multifactorial but the role of intervertebral disc (IVD) degeneration *per se *in LBP is becoming clearer [3]. Imaging studies indicate a link between IVD degeneration and LBP [3,4], with the most clinically significant correlations between degenerate disc space narrowing (which develops as degeneration progresses [5,6]) and chronic LBP [7]. A key target for the treatment of LBP is therefore the restoration of disc height, which could be achieved via the regeneration of the extracellular matrix in the degenerate IVD.

Evidence from studies investigating the pathogenesis of IVD degeneration illustrates that IVD degeneration originates from the nucleus pulposus (NP), where both type II collagen and proteoglycan synthesis and content decrease [8,9], thus the NP is the area of the disc that is targeted by a number of groups worldwide for regeneration strategies [10,11]. Numerous methods are under investigation to stimulate regeneration of the disc, including growth factor treatments and cell-based therapies which either utilize cells alone or combined with scaffolds [12]. A cell source that has been suggested for such therapies is that of autologous disc cells harvested from adjacent nondegenerate discs, although removal of cells from a donor disc can induce degeneration, and thus would be unsuitable [13]. Other studies have suggested using autologous degenerate IVD cells extracted during discectomy, which following *in vitro *expansion would be reinserted into the degenerate IVD [14,15]. We have previously shown, however, that cells derived from a degenerate IVD show a senescent phenotype [16,17], which results in a reduced cell replication potential, and thus the expansion capabilities of degenerate IVD cells are limited. Furthermore we have shown that IVD cells derived from a degenerate disc display an abnormal phenotype, with increased catabolic and decreased anabolic activity, and thus are not the ideal cell type to stimulate regeneration, and indeed could even lead to accelerated degeneration of the treated IVD [18-24].

An alternative cell source are adult stem cells, in particular, bone-marrow-derived mesenchymal stem cells (MSCs). The use of these cells would allow an autologous approach, reducing the risk of rejection and infection. MSCs are multipotent, and have the ability to differentiate into an NP-like phenotype when appropriately stimulated [25-29]. To date, however, the optimum method of application of these cells for repair/regeneration strategies for the IVD is unclear. A number of studies have described the development of tissue-engineered gels and scaffolds seeded with MSCs to assist in the regeneration of the IVD [10], and have shown promising results *in vitro*. Yet it is unclear whether a scaffold would be required to assist in tissue regeneration or whether the *in vivo *tissue niche and/or local cells alone are sufficient to stimulate appropriate MSC differentiation.

Work from our laboratory has shown that co-culture of MSCs with NP cells *in vitro *is capable of inducing differentiation to an NP-like phenotype [26]. This raises the possibility that the native IVD cells *in vivo *could induce MSC differentiation without the need for external manipulation. Such an approach would be of great benefit for mild and moderate stages of degeneration and could also be useful as a preventative strategy following disc surgery to adjacent IVDs to prevent the accelerated degeneration often seen within these discs. A recent study by Ho and colleagues also suggests that MSC injection therapies may show potential at late stages of degeneration [30]. Treatment at this stage would, however, in all likelihood require some form of combined therapy utilising an appropriate scaffold to provide support to the cells and restore IVD height immediately whilst the matrix is formed. Additionally, such strategies would probably require combined treatments to restrain the degenerative processes – such as inhibition of IL-1, which is significantly increased in IVD degeneration and has been shown to be involved in matrix degradation [18,31].

Interestingly only a limited number of studies have investigated the injection of MSCs into IVDs, and, although these have demonstrated cell survival and increased proteoglycan (PG) production within the IVD [28,30,32-36], few have investigated the phenotype of the injected stem cells. Sakai and colleagues investigated the injection of rabbit MSCs, within an aetocollagen gel, into the rabbit disc, and demonstrated that the MSCs differentiated to a chondrocyte-like phenotype and increased the collagen and PG content within the disc [28]. Whether the carrier gel was responsible for inducing MSC differentiation, however, was unclear. More recently Hiyama and colleagues directly injected MSCs into a canine degenerate disc model; although the study tracked the MSCs, only Fas ligand expression and overall PG production was assessed and the authors did not determine whether the injected MSCs displayed appropriate differentiation and phenotype [36]. Importantly, no studies to date have investigated the phenotype of human MSCs following injection into IVD tissue.

In the present article we established an *in vitro *model system to investigate the survival and phenotype of human MSCs following injection into bovine NP tissue explants to test the following hypothesis: that the IVD tissue niche itself can induce the differentiation of MSCs to a disc-like phenotype and direct the cells to form a new and appropriate matrix.

## Materials and methods

### Mesenchymal stem cell source and extraction

Bone marrow samples were received from two patients (aged 66 and 74 years) undergoing hip replacements. Informed consent from the patients and local ethical committee approval were obtained. Bone marrow was negatively sorted for haematopoietic cells using RosetteSep (STEMCELL Technologies SARL, Grenoble, France) prior to isolation of mononuclear cells using a Histopaque 1077 gradient (Sigma, Poole, UK). Cells were cultured for 7 days and any nonadherent cells were removed. MSCs (characterised by their adherence to plastic and morphology) were then expanded in a monolayer and used at low passage (passage < 2). The multipotentiality of these MSCs was assessed via differentiation along the three common mesenchymal lineages (osteogenic, adipogeneic and chondrogenic) using standard methodology.

### Nucleus pulposus tissue explant culture

Bovine tails from 9-month-old to 18-month-old cows were obtained from the abattoir. Caudal IVDs were excised and NP tissue was isolated. Cores of NP tissue (0.5 cm in diameter and 0.6 cm high) were formed and placed into a Perspex ring culture system as described previously [37]. DMEM + F12 media supplemented with 10% v/v heat-inactivated FCS (Gibco, Paisley, UK), 100 U/ml penicillin (Sigma, Poole, UK), 100 μg/ml streptomycin (Sigma), 250 ng/ml amphotericin, 2 mM glutamine (Sigma) and 50 μg/ml ascorbic acid (Sigma) (complete cell culture media) was applied and tissue explants were maintained in culture for 1 week prior to MSC injection.

### Cell labelling

To allow cell tracking following cell injection, the MSCs were infected with adenoviral vectors carrying the green fluorescent protein transcript (Ad-GFP). The optimal multiplicity of infection was determined as 1,000, which resulted in 100% infectivity without cytotoxic effects (data not shown). To perform infection, MSCs in a monolayer culture were typsinised from flasks, cell counts were performed and then cells were re-seeded into T75 flasks in 5 ml complete media and were allowed to adhere for 4 hours. Following adherence of MSCs, an appropriate volume of Ad-GFP (Vector Biolabs, Philadelphia, PA, USA) was applied to achieve a multiplicity of infection of 1,000 and was left for 2 hours to allow initial infection. Thereafter, 10 ml fresh complete media was applied to each flask and left for 72 hours for viral transfer to occur as previously published [31].

### MSC transfer to nucleus pulposus tissue explants

Ad-GFP-infected MSCs were trypsinised in 1× trypsin/ethylenediamine teraacetic acid (Invitrogen, Paisley, UK) and inactivated in complete media, and cell counts were performed. Cells were centrifuged at 400 × *g *for 10 minutes and were resuspended in complete media at a cell density of 2 × 10^6 ^cells/ml. An aliquot of Ad-GFP-infected MSCs was visualised using fluorescent microscopy (450 nm excitation) to ascertain the infection efficiency. The media was removed from NP tissue explants and 50 μl Ad-GFP-infected MSC suspension (that is, 100,000 cells) was injected into tissue explants while 50 μl media containing no cells was injected into control tissue explants. Such a cell number equates to an extra 1,178 cells/mm^3^, which is approximately one-quarter of the cell density reported for normal human NP (4,000 cells/mm^3^) [38] and thus should be maintainable *in vivo*. Ten millilitres of complete media was then applied to each tissue explant, and the explants were cultured for up to 4 weeks and the media changed every 2 to 3 days. Duplicate tissue samples (that is, two control explants; two explants injected with MSC sample 1; and two explants injected with MSC sample 2) were removed at 48 hours, 1 week, 2 weeks and 4 weeks post injection.

### Processing of tissue explants and identification of the injection site

Tissue explants were fixed in 4% w/v paraformaldehyde/PBS overnight prior to routine paraffin embedding. Tissue samples were serially sectioned at 4 μm, and one section every 80 μm was mounted onto positively charged slides (Thermo Shandon, Fife, Scotland, UK). Sections were air-dried, dewaxed in xylene, dehydrated in industrial methylated spirit, air-dried, and mounted in immersion oil (Sigma) and were viewed using fluorescent microscopy to identify green fluorescent protein (GFP)-infected cells. Following identification of the position of injection site and the presence of GFP-labelled cells, serial sections in the area of the injection site were mounted onto positively charged slides: for *in situ *hybridisation for polyA-mRNA to assess cell metabolic activity; for immunohistochemistry for caspase 3 to identify the presence of apoptotic cells; for immunofluorescence and immunohistochemistry for aggrecan, Sox-9 and types I, II and X collagen to assess phenotypic characteristics; and for histochemistry with Alizarin red to assess mineralisation.

### *In situ *hybridisation for polyA-mRNA

*In situ *hybridisation for polyA-mRNA was performed as an assessment of cell metabolic activity as described previously [39,40].

### Immunofluorescence and immunohistochemistry

Immunofluorescence and immunohistochemistry were both performed for aggrecan, Sox-9, and types I, II and X collagen, and immunohistochemistry was performed for caspase 3 as described previously [22]. Briefly, 4 μm paraffin sections were dewaxed, rehydrated and endogenous-peroxidase-blocked using hydrogen peroxide. After washing in dH_2_O, sections were then treated with the required antigen retrieval system (Table 1). Following washing, nonspecific binding sites were blocked at room temperature for 45 minutes with appropriate serum, and sections were incubated overnight at 4°C with primary antibodies (Table 1). Negative controls in which mouse IgG, rabbit IgG or goat IgG (Dako, Ely, Cambridgeshire, UK) replaced the primary antibody (at an equal protein concentration) were used. After washing, sections were reacted with secondary antibodies (Table 1) for 30 minutes at room temperature. Disclosure of antibodies was performed by immunofluorescence and immunohistochemistry.

**Table 1:** Details of the immunohistochemistry methodology employed

Target	Antigen retrieval	Blocking step and primary antibody	Secondary antibody
Aggrecan	0.1% w/v hyaluronidase in Tris-buffered saline (Sigma, Poole, UK), 30 minutes at 37°C	20% v/v rabbit serum, and mouse monoclonal aggrecan 1° (1:25 dilution; AbCam, Cambridge, UK)	Biotinylated rabbit anti-mouse antiserum (1:400; Dako, Ely, Cambridgeshire, UK)
Sox-9	None required	20% v/v swine serum, and rabbit polyclonal Sox-9 1° (1:100 dilution; SantaCruz, Heidelburg, Germany)	Biotinylated swine anti-rabbit antiserum (1:400; SantaCruz)
Type I collagen	0.01% hyaluronidase (Sigma), 0.02% trypsin (Sigma) w/v in Tris-buffered saline	20% v/v rabbit serum, and mouse monoclonal type I collagen 1° (1:250 dilution; ICN, Basingstoke, UK),	Biotinylated rabbit anti-mouse antiserum (1:400; Dako)
Type II collagen	0.1% w/v hyaluronidase in Tris-buffered saline (Sigma), 30 minutes at 37°C	20% v/v rabbit serum, and mouse monoclonal type II collagen 1° (1:100 dilution; MP Biomedicals, Illkirch, France)	Biotinylated rabbit anti-mouse antiserum (1:400; Dako)
Type X collagen	1 mg/ml hyaluronidase, 0.25 U/ml chondrotinase in Tris-buffered saline, 1 hour at 37°C; followed by 0.1% protease in Tris-buffered saline, 10 minutes at 37°C	25% v/v goat serum, and mouse monoclonal type X collagen 1° (1:200 dilution; AbCam)	Biotinylated goat anti-mouse antiserum (1:100; SantaCruz)
Caspase 3	None required	20% v/v donkey serum, and goat polyclonal caspase 3 1° (1:500 dilution; SantaCruz)	Biotinylated donkey anti-goat antiserum (1:300; SantaCruz)

### Immunofluorescence detection

Disclosure of secondary antibody binding was performed by incubation in 1:50 dilution of rhodamine-conjugated Biotin (Jackson ImmunoResearch, Newmarket, Suffolk, UK) for 1 hour at room temperature. Following washes, sections were counterstained with 4',6-diamidino-2-phenylindole for 10 minutes, air-dried, mounted in immersion oil and viewed immediately.

### Immunohistochemical detection

Disclosure of secondary antibody binding followed the streptavidin-biotin complex (Dako) technique with 3,3'-diaminobenzidine tetrahydrochloride solution (Sigma). Sections were counterstained with Mayers haematoxylin (Raymond A Lamb, Eastborne, East Sussex, UK), dehydrated and mounted in XAM (BDH, Poole, UK).

### Image analysis

All slides were visualised using a Leica RMDB research microscope (Leica Biosystems Peterborough Ltd, Peterborough, UK) and images were captured using a digital camera and Bioquant Nova image analysis system (BIOQUANT Image Analysis Corporation, Nashville TN, USA). Immunofluorescence images were viewed under a fluorescent microscope with filters for 4',6-diamidino-2-phenylindole (420 to 495 nm), GFP (510 to 560 nm) and rhodamine (663 to 738 nm). Images were captured within each sample to qualitatively analyse the injection site and native disc cells and matrix. Image capture for all three wavelengths on the same field of view was performed to enable identification of GFP-positive cells and immunopositivity for matrix proteins in the same cells.

## Results

### Identification of injected mesenchymal stem cells

**Figure 1:**
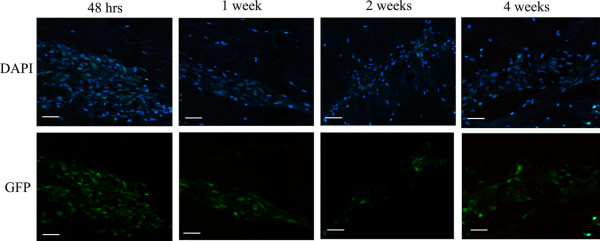
Identification of injected green fluorescent protein adenoviral vector infectedmesenchymal stem cells in nucleus pulposus tissue explants. Photomicrographs of 4',6-diamidino-2-phenylindole (DAPI) staining and green fluorescent protein (GFP)-positive cells in the injection sites of intervertebral disc tissue at 48 hours, 1 week, 2 weeks and 4 weeks post injection of mesenchymal stem cells infected with adenoviral vectors carrying the GFP transcript. Scale bar = 570 μm.

No GFP-positive cells were observed within control tissue in which no MSCs had been injected, demonstrating that native disc tissue did not autofluoresce. Ad-GFP-labelled MSCs were identified in all tissue samples in which Ad-GFP-infected MSCs were injected and cells were observed in the vicinity of the injection site at all time points post injection. At 4 weeks post injection some Ad-GFP MSCs appeared to have migrated into the tissue away from the injection site, but the majority of the MSCs remained in a cellular cluster within the injection site (Figure 1).

### Cell viability/metabolic activity

**Figure 2:**
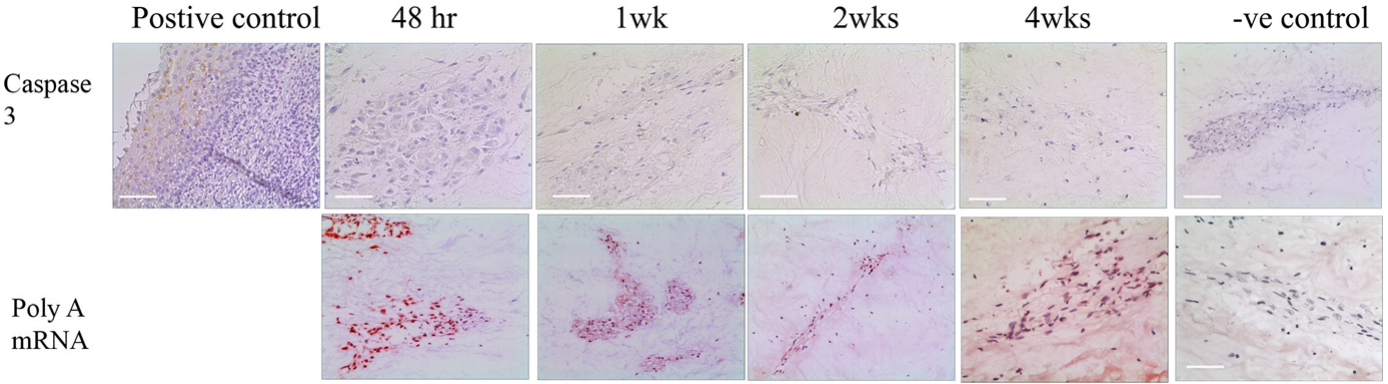
Cell viability/metabolic activity of injected mesenchymal stem cells. Photomicrographs representative of caspase 3 immunopositivity and polyA-mRNA staining in mesenchymal stem cells injected into tissue explants at 48 hours, 1 week, 2 weeks and 4 weeks post injection. Scale bar = 570 μm.

Few apoptotic bodies were observed within GFP-labelled injected MSCs in all tissue samples. Low levels of apoptosis were confirmed with immunohistochemistry for caspase 3, which was performed on multiple sections throughout the depth of injection site. At the site of injection no caspase-3-immunopositive cells were observed at 48 hours post injection. A small number of caspase-3-immunopositive cells, however, were observed at 1 week (average 6.98%) and 2 weeks (average 14.23%) post injection. No caspase-3-positive cells were seen following 4 weeks post injection (Figure 2a). IgG controls were negative (Figure 2a). No caspase 3 staining was observed in any of the native disc cells at any time point. *In situ *hybridisation for polyA-mRNA demonstrated that MSCs injected into tissue explants showed higher levels of metabolic cell activity than the native cells, particularly 48 hours post injection. Cell activity identified by red cell staining was maintained for the 4 weeks investigated (Figure 2b). Negative controls (that is, no probe) did not display any positive staining (Figure 2b).

### Matrix protein expression and formation by native disc cells

**Figure 3:**
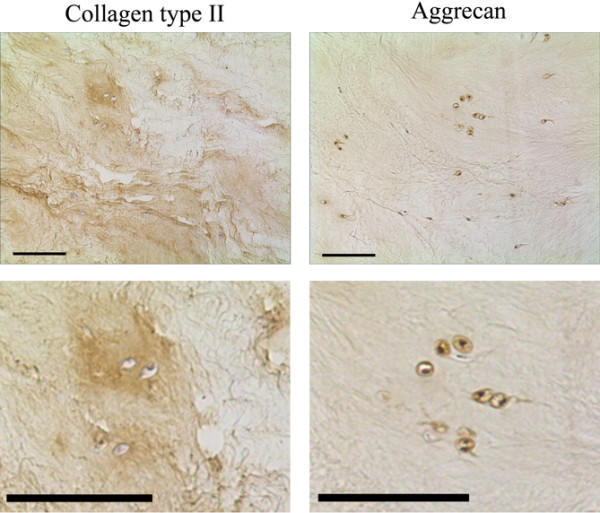
Phenotypic characteristics of native disc cells. Photomicrographs representative of type II collagen and aggrecan immunohistochemical staining in control (that is, noninjected) tissue explants of nucleus pulposus tissue. Scale bar = 570 μm.

Native disc cells displayed immunopositive staining (assessed by both immunofluorescence and 3,3-diaminobenzidine (DAB) disclosure) for Sox-9, type II collagen and aggrecan. In addition, matrix staining was observed for type II collagen and aggrecan (Figure 3). No immunopositivity was observed for type I collagen or type X collagen.

### Matrix protein expression and formation by injected MSCs

**Figure 4:**
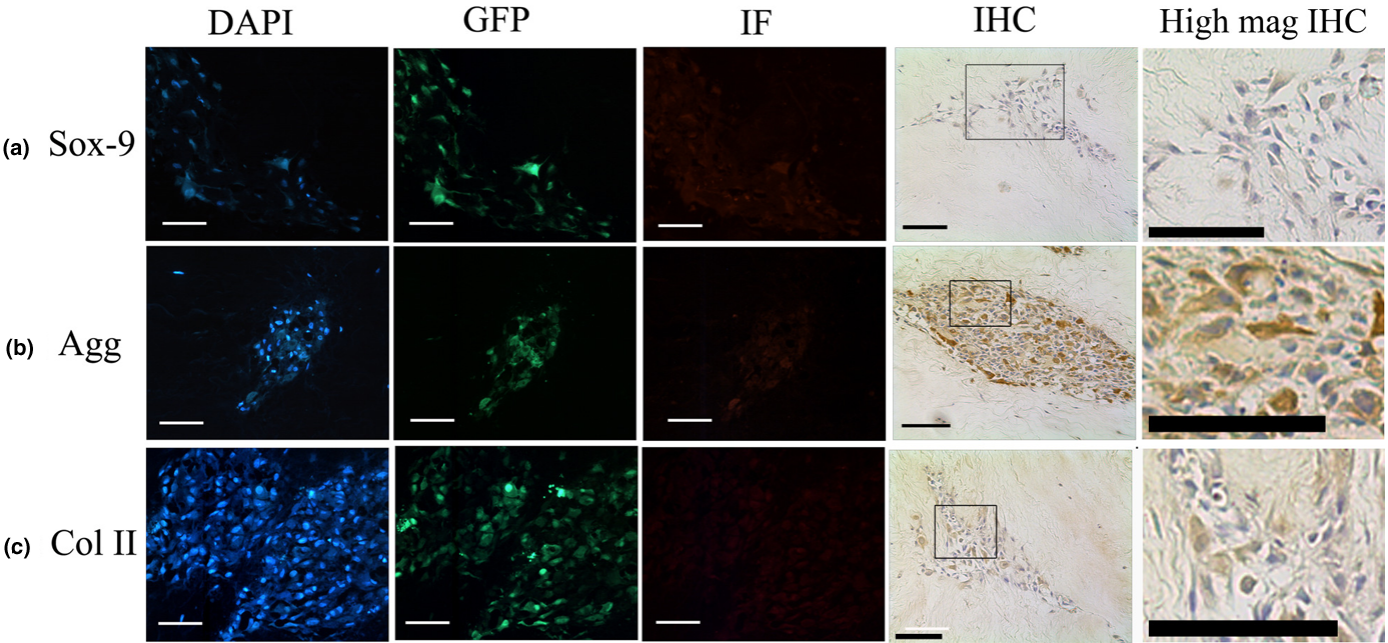
Phenotypic characteristics of injected mesenchymal stem cells following 48 hours in culture. Photomicrographs representative of 4',6-diamidino-2-phenylindole (DAPI) counterstaining, green fluorescent protein (GFP) localisation and immunofluorescence (IF) for identical field of view and immunohistochemistry (IHC) in tissue injected with mesenchymal stem cells and cultured for 48 hours post injection: **(a) **Sox-9, **(b) **aggrecan (Agg) and **(c) **type II collagen (Coll II). High Mag IHC, high magnification of indicated IHC region. Scale bar = 570 μm.

**Figure 5:**
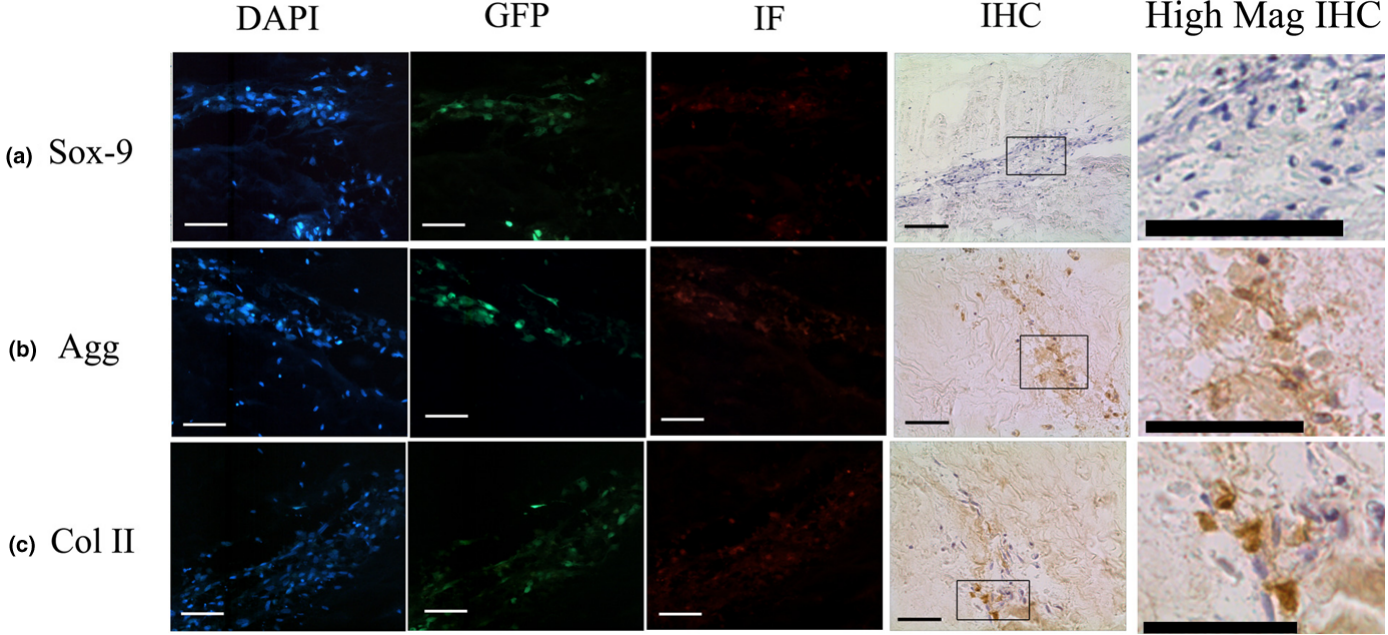
Phenotypic characteristics of injected mesenchymal stem cells following 4 weeks in culture. Photomicrographs representative of 4',6-diamidino-2-phenylindole (DAPI) counterstaining, green fluorescent protein (GFP)localisation and immunofluorescence (IF) for identical field of view and immunohistochemistry (IHC) in tissue injected with mesenchymal stem cells and cultured for 4 weeks post injection: **(a) **Sox-9, **(b) **aggrecan (Agg) and **(c) **type II collagen (Coll II). High Mag IHC, high magnification of indicated IHC region. Scale bar = 570 μm.

Immunohistochemistry was used to assess the expression and localisation of the chondrogenic transcription factor Sox-9, and of the matrix genes aggrecan and types I, II and X collagen within MSCs both in the monolayer and in those injected into NP tissue explants. Monolayer MSCs displayed no Sox-9 immunopositivity, but upon injection into NP tissue explants MSCs were immunopositive (as assessed by immunofluorescence and 3,3-diaminobenzidine disclosure) for Sox-9 at 48 hours and 1 week post injection (Figure 4a). No immunopositivity for Sox-9 in these injected cells, however, was observed 2 weeks or 4 weeks post injection (Figure 5a).

Monolayer MSCs showed no immunopositivity for aggrecan. Following injection into IVD tissue explants, however, MSCs were immunopositive (assessed both by immunofluorescence and 3,3-diaminobenzidine disclosure) for aggrecan and the staining intensity increased with time post injection (Figures 4 and 5b). Aggrecan matrix staining within the vicinity of the injected cells was also observed 1 week following injection, and the intensity of the matrix staining increased with time in culture (Figures 4 and 5b).

Weak staining for type II collagen was observed within a small number of cells in monolayer culture. Following injection into NP tissue explants, MSCs stained strongly positive for type II collagen and matrix staining in the vicinity of the injected MSCs was also observed 1 week post injection (Figure 4b). Both MSC cell and matrix staining for type II collagen increased with time in the explant culture (Figure 5b).

Type I collagen cell and matrix staining was observed in MSCs in the monolayer culture. Following injection of MSCs into NP tissue explants, type I collagen matrix staining was observed in close proximity to the injection site, although this decreased with time in culture. No cellular immunopositivity for type I collagen or type X collagen was observed in MSCs injected into NP tissue cultures at any time point. IgG controls were negative at all time points. Alizarin red staining was performed to assess mineralisation, and no positive staining was observed either in the MSCs or in native tissue during the culture period investigated.

## Discussion

The decreased disc height characteristic of IVD degeneration has often been linked to LBP [3], and thus regeneration strategies aimed at restoring the disc extracellular matrix and restoring disc height have been postulated as potential treatments. One such therapy under investigation by a number of groups worldwide is the use of autologous MSCs to aid in the regeneration of the IVD extracellular matrix. To date, however, the optimum method of application of these cells for regeneration strategies for the IVD is unclear, and few studies have investigated the direct injection of MSCs alone into IVD tissues.

In the present article we investigated the survival and phenotype of human MSCs sourced from aged osteoarthritic hips following injection into NP tissue explant cultures. The supply of autologous MSCs used in cell-based therapies for regeneration of the degenerate IVD would probably be sourced from older individuals similar to those used within this study as the incidence of disc degeneration increases with age [41]. Furthermore, MSCs sourced from aged and arthritic hips represents the poorest cell source for MSCs as these cells have been suggested to have a tendency for osteogenic differentiation [42], which would be detrimental for the repair of the IVD. Our study, however, demonstrated no type X collagen formation or mineralisation in the IVD tissue 4 weeks post injection. The finding that such cells not only survive following injection into IVD tissue but appear to redifferentiate into a chondrocyte-like phenotype, typical of an NP cell, without any induction of mineralisation is therefore of paramount importance for future autologous cell-based therapies.

Crevensten and colleagues injected rat MSCs within a viscous hyaluronan gel into rat IVDs *in vivo*, and demonstrated a loss in cell number between 1 and 7 days in culture [35] – indicating high levels of cell death, which the authors attributed to carrier gel toxicity. In the present study we demonstrated good cell viability at all time points post injection with little cell death as evidenced by caspase 3 immunopositivity or the presence of apoptotic bodies. The improved cell viability observed may be the result of the injection method as cells were directly injected into the tissue rather than seeding into a gel prior to insertion, or may alternatively be a result of the *in vitro *culture conditions in that there would be higher nutrient supply than that *in vivo*. Zhang and colleagues showed no change in MSC cell number between 1, 3 and 6 months post injection, suggesting good cell viability of rabbit MSCs injected into rabbit discs [34], and Hiyama and colleagues suggested that MSCs following injection into canine discs appeared to proliferate and have good survival rates [36].

Zhang and colleagues demonstrated an increase in aggrecan and type II collagen following 1, 3 and 6 months post injection of rabbit MSCs into rabbit discs *in vivo *compared with noninjected tissues. Unfortunately, however, no localisation studies were performed to determine whether this increase resulted from the injected MSCs or from increased synthesis of aggrecan and type II collagen by the native disc cells [34]. In the present study, however, we have demonstrated that cellular protein expression and local matrix accumulation for aggrecan and type II collagen was observed within the MSCs following injection into disc tissue. This suggests that the IVD tissue niche within the *in vitro *system studied here results in the differentiation of the injected MSCs to a chondrocyte-like phenotype, typical of an NP cell. An *in vivo *study also demonstrated that MSCs transplanted into a rabbit IVD displayed an NP-like phenotype with expression of proteoglycans and type II collagen at 2 weeks post transplantation [28], although in that study it was unclear whether the carrier aetocollagen gel aided differentiation.

Interestingly, our results would appear to suggest that the increased proteoglycan and collagen production observed in a number of *in vivo *studies following injection of MSCs into disc tissue [28,34,36] may be due to differentiation of the MSCs to a chondrocyte-like phenotype, induced by the local IVD tissue niche/native cells. The effect of the IVD tissue niche on injected MSCs could be due to the close proximity of the MSCs with native disc cells, as co-culture of MSCs with NP cells has been shown to induce the differentiation of MSCs to an NP-like phenotype in monolayer and pellet culture systems *in vitro *[26,29]. Alternatively the availability of growth factors such as transforming growth factor beta (which has been shown to assist in MSC differentiation to an NP-like phenotype [43,44]) sequestered in the IVD matrix may direct MSC differentiation. The most probable scenario, however, is that the IVD tissue niche composed of the native cells, matrix, and growth factors all play a role in the differentiation of the MSCs post injection.

Our work together with the data provided by others is promising for successful future therapeutic use of MSCs in that it suggests spontaneous differentiation of MSCs into an NP-like cell following insertion into the disc. The results of our study importantly show that this differentiation occurs without the need for additional stimuli such as that provided by a carrier gel or additional growth factor treatments. There are limitations to our study, however, which must be considered when extrapolating these data to repair of the degenerate human disc *in vivo*. The culture conditions used here do not mimic that of the human degenerate IVD, where the cells are exposed to a hypoxic, low-nutrient and mechanically loaded environment. These factors could of course alter the behaviour of the injected MSCs and may affect their differentiation in the disc. In addition, the degenerate disc is a hostile environment with increased production of cytokines that alter matrix synthesis and expression of matrix degrading enzymes [19,21-23]. These cytokines may influence MSC differentiation and subsequent behaviour, and thus in such a situation a combined therapy where these degenerative processes are also inhibited may be required [18,31]. Interestingly, however, Sakai and colleagues demonstrated promising results in a rabbit model of degeneration where they showed enhanced matrix formation [33] following injection of MSCs embedded in atelocollagen. Our current study utilising an *in vitro *model system suggests that a simpler approach utilising direct injection of MSCs into the disc could induce regeneration of the disc via differentiation of injected MSCs and subsequent formation of new and appropriate matrix. The key advantages of this technique would be that such an approach reduces the cost, the risk of infection and the time between MSC cell harvest and cell therapy.

Importantly the development of the present *in vitro *model to test the survival, phenotype and function of human MSCs following injection into IVD tissue is a major advance for testing the efficacy of future therapies. This culture system can be utilised to investigate MSC behaviour in human IVD tissue samples from both nondegenerate and, importantly, degenerate tissue that would not be possible *in vivo*. This *in vitro *system also allows the manipulation of the local environment in a controlled manner to study factors such as reduced oxygen, nutrients or the influence of load on the phenotype and survival of injected MSCs. All of these are important questions to address before clinical use of MSC therapies becomes a reality – and the development of the *in vitro *system described here, in which MSCs can be tracked and their phenotype/function assessed under such conditions, will allow these studies to be conducted.

## Conclusion

Using an *in vitro *model system we have shown that MSCs differentiate spontaneously upon insertion into NP tissue and thus may not require additional stimulation or carrier to induce differentiation. This is a key finding because such a strategy would minimise the level of external manipulation of the MSCs required prior to insertion into the patient, thus simplifying treatment strategy and reducing costs. Future studies will involve the investigation of the behaviour of these cells following injection into degenerate human IVD tissue explants, and the influence of a loaded, hypoxic and low-nutrient environment (mimicking the human *in vivo *milieu) on cell survival and differentiation.

## Abbreviations

Ad-GFP: adenoviral vectors carrying the green fluorescent protein transcript; DMEM: Dulbecco's modified Eagle's medium; FCS: foetal calf serum; GFP: green fluorescent protein; IL: interleukin; IVD: intervertebral disc; LBP: low back pain; MSC: mesenchymal stem cell; NP: nucleus pulposus; PBS: phosphate-buffered saline; PG: proteoglycan.

## Competing interests

The authors declare that they have no competing interests.

## Authors' contributions

CLLM helped conceive the study, helped to secure funding, participated in its design, performed the majority of the laboratory work and all of the analysis, and co-wrote the manuscript. PB performed the type X collagen and Alizarin red staining, and participated in interpretation of the data. AJF participated in interpretation of the data and contributed to the preparation of the final manuscript. JAH conceived the study, secured funding, contributed to its design and coordination, participated in interpretation of the data and co-wrote the manuscript. All authors read and approved the final manuscript.
